# Microstructural characteristics of the stony coral genus *Acropora* useful to coral reef paleoecology and modern conservation

**DOI:** 10.1002/ece3.7247

**Published:** 2021-03-09

**Authors:** Meixia Zhao, Haiyang Zhang, Yu Zhong, Xiaofeng Xu, Hongqiang Yan, Gang Li, Wen Yan

**Affiliations:** ^1^ Key Laboratory of Ocean and Marginal Sea Geology South China Sea Institute of Oceanology Innovation Academy of South China Sea Ecology and Environmental Engineering Chinese Academy of Sciences Guangzhou China; ^2^ Southern Marine Science and Engineering Guangdong Laboratory (Guangzhou) Guangzhou China; ^3^ University of Chinese Academy of Sciences Beijing China; ^4^ Daya Bay Marine Biology Research Station Chinese Academy of Science Shenzhen China

**Keywords:** *Acropora*, Indo‐Pacific, microstructure, Scleractinia, South China Sea, taxonomic identification, thin section

## Abstract

Identification of fossil corals is often limited due to poor preservation of external skeleton morphology, especially in the genus *Acropora* which is widespread across the Indo‐Pacific. Based on skeleton characteristics from thin section, we here develop a link between the internal skeleton structure and external morphology. Ten characteristics were summarized to distinguish *Acropora* and five related genera, including the type and differentiation of corallites, the skeleton nature of corallites (septa, columellae, dissepiments, wall), and calcification centers within septa. *Acropora* is distinctive for its dimorphic corallites: axial and radial. *Isopora* is similar to *Acropora* but possess more than a single axial corallites. *Montipora* and *Astreopora* (family Acroporidae) have monomorphic corallites and a synapticular ring wall, with clustered calcification center in the former and medial lines in the latter. *Pocillopora* and *Porties* are classified by distinctive dissepiments, columellae and septa. These microstructural skeleton characteristics were effective in the genus identification of fossil corals from drilled cores in the South China Sea. Eighteen detailed characteristics (ten of axial corallites, four of radial corallites, and four of coenosteum) were used in the *Acropora* species classification. The axial corallites size and structure (including corallite diameter, synapticular rings, and septa), the septa of radial corallites, and the arrangement of coenosteum were critical indicators for species identification. This identification guide can help paleoenvironmental and paleoecological analyses and modern coral reef conservation and restoration.

## Graphical abstract

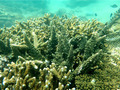

## 1 INTRODUCTION

Coral reefs are highly diverse and have existed for a long time (Stanley et al., 2018; Veron et al., 2015). They are of great ecological and socioeconomic importance, but are subject to recent dramatic declines as a consequence of both natural and anthropogenic disturbances (Burke et al., 2011; Fine et al., 2019; Zhao & Yu, 2006). Global‐scale effects by climate change combine with local‐level impacts as severe stressors on coral reefs (Carpenter et al., 2008; Hoegh‐Guldberg et al., 2017; Riegl et al., 2009). To better understand changes in present‐day and future reef ecosystems due to climate change and other human activities, it is helpful to establish baselines from paleoecological records (Hongo et al., 2017; Perry et al., 2008; Ryan et al., 2016). While heavily impacted and increasingly degraded now, coral reefs have been resilient to past sea‐level and temperature fluctuations over long timescales (Greer et al., 2009; Webster et al., 2018). Therefore, understanding the development of ancient coral reefs and their responses to natural environmental change is helpful to aid protection of presently healthy reefs and to restore degraded reefs in future (Humblet & Webster, 2017; Kuffner & Toth, 2016; Odea et al., 2020).

Scleractinian corals are key for maintenance of biodiversity and ecological function of coral reefs. The genus *Acropora* reaches its zenith in the modern coral communities of the Indo‐Pacific (Wallace, 2012). Rapidly growing branching *Acropora* have contributed to reef formation from the late Oligocene (28–23 Ma) to present (Wallace & Rosen, 2006; Wilson et al., 2019). Since many *Acropora* species are sensitive to the impact of coral bleaching due to elevated sea temperatures (Hughes et al., 2018; Morrison et al., 2019) and other damage from anthropogenic exploitation and disturbance (Fabricius, 2005; Wilkinson, 2008), the future persistence and ecological function of *Acropora* in the current scenario of rapid global climate change is of great concern (Carpenter et al., 2008; Hughes et al., 2018; Perry et al., 2013, 2018). The remarkable resilience of *Acropora* corals to the large‐scale climate and environmental changes over the historical period was demonstrated from fossil records of ancient reefs (Humblet & Webster, 2017; Webster et al., 2018; Webster & Davies, 2003). For example, *Acropora* thrived across the Holocene Thermal Maximum in the Caribbean and the Persian Gulf, and its decline over the past decades is due to unprecedented ecological changes related to anthropogenic activity (Greer et al., 2009; Samimi‐Namin & Riegl, 2012; Wapnick et al., 2004). Therefore, the fossil *Acropora* record is important for paleoenvironmental/ecological studies, and may provide valuable information for conservation and restoration of modern coral reefs (Samimi‐Namin & Riegl, 2012).

The earliest occurrences of *Acropora* in the fossil record dates from the Paleocene of Somalia (Carbone et al., 1994) and Austria (Baron‐Szabo, 2006). Fossils revealed a high diversity of staghorn *Acropora* since the Neogene (Santodomingo et al., 2015; Wallace, 2012; Wallace & Bosellini, 2015). The taxonomic identification of fossil *Acropora* corals is often limited to generic level because of its poor preservation and the fossils missing many morphological features (Humblet et al., 2015; Ryan et al., 2016; Webster & Davies, 2003). Traditional classification of scleractinian corals is mainly based on skeletal morphology (macromorphology: the size and shape of many features related to corallite architecture and the integration of corallites within colonies; micromorphology: the shapes of teeth and granules along the margins and faces of septa; microstructure: the arrangements of centers and fibers within the wall, septa, and columella). Macromorphological characters are important at the generic and specific levels, whereas micromorphological characters at the familial level and above (Wells, 1956). With the exception of Alloiteau (1957) and Chevalier and Beauvais (1987), microstructural characters are less commonly used in traditional taxonomy (Budd et al., 2010). With the recent advances in scleractinian taxonomy, the microstructure of skeletons linking present‐day corals with fossil species is becoming more important (Budd & Stolarski, 2009, 2011). Detailed revisions of the families Mussidae, Merulinidae, Montastraeidae, Diploastraeidae, and Lobophylliidae examined coral skeletal features at three distinct levels (Budd et al., 2012; Huang et al., 2014, 2016).

Since many coral species that inhabit modern reef ecosystems have no significant morphological changes since the Oligocene–Miocene extinction event (Stanley, 2003), the identification of Cenozoic fossil corals is based on the same criteria used to identify modern corals despite the limited exposure of external features in the fossil record. Most fossil *Acropora* are collected from outcrops or drilled cores, and most external morphological features are missing or only cross sections are available for identification. The microstructure of skeletons usually preserves adequate structural details. A comprehensive taxonomic review of *Acropora* species using 23 macro‐ and micromorphological characters defined 20 “species groups” (Wallace, 1999). The microstructural characters reflected from petrograpic thin sections can now be summarized and added to the process of fossil *Acropora* identification.

**FIGURE 1:**
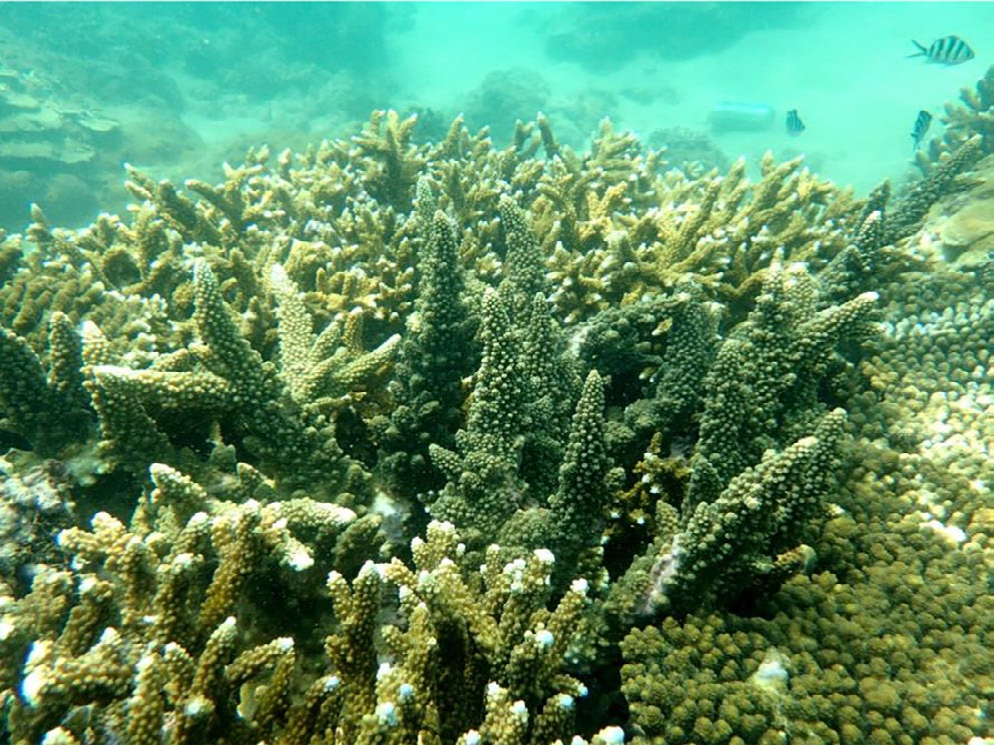
Coral community in the South China Sea. This picture was taken from the coral reef of Hainan Island in 2017

The South China Sea is the largest marginal sea in the Indo‐Pacific, and it covers an area over 3 million km^2^ (Morton & Blackmore, 2001). Coral reefs and atolls occur abundantly over an area of at least 8,000 km^2^ (Yu, 2012) with a long evolutionary history (Fan et al., 2019; Wang & Li, 2009; Wang et al., 2018). Despite containing only 17% of the reef area as the Coral Triangle, this region possesses extraordinary coral biodiversity and could rivals that of Coral Triangle. Among 571 known species of reef corals recorded in the South China Sea, 98 are *Acropora*, some of which are dominant and key species for their contribution to coral cover and reef habitat construction (Huang et al., 2015; Zhao et al., 2013, 2017; Figure 1).

We collected living coral specimens from modern coral reefs and (sub)fossil coral in drilled cores from the South China Sea, to analyze the microstructure of skeletons of *Acropora* and other related genera. The aims of this paper are (a) to generalize the microstructural skeleton characteristics of living *Acropora* and five related coral genera on genus level; (b) to describe the detailed microstructural skeleton characteristics of living Acropora at species level; (c) to complete identification of fossil *Acropora* and related genera from drilled cores according to their microstructural features; and (d) to show the relevance of this process to fossil *Acropora* species identification. We provide microstructure characteristics useful to identification of both extant and fossil *Acropora*. This work will be helpful for paleoenvironmental and paleoecological analyses and modern coral reef conservation and restoration.

## 2 MATERIALS AND METHOD

### 2.1 Coral sampling

**FIGURE 2:**
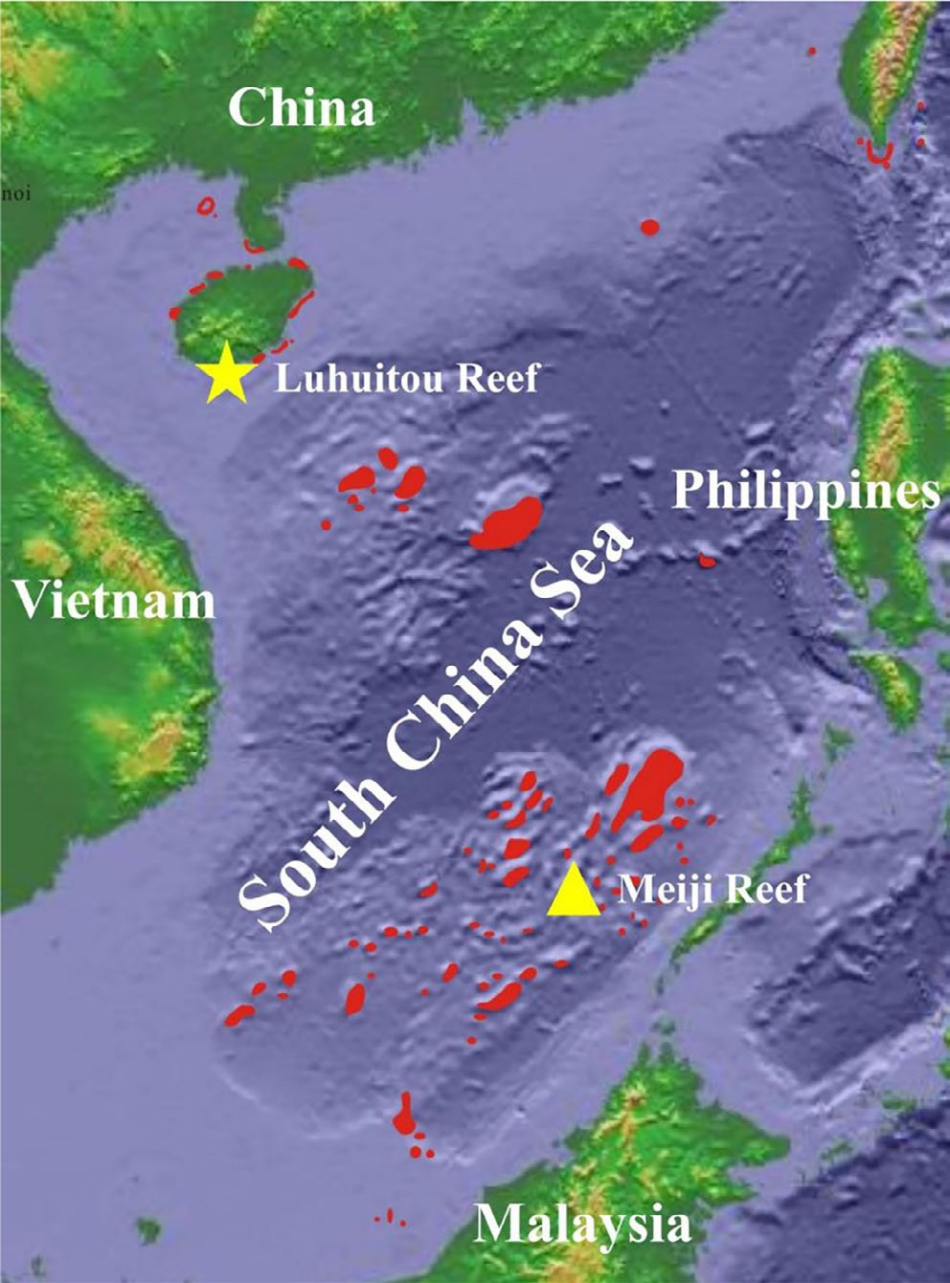
Distribution map of reefs in the South China Sea and sample sources (yellow pentagon represents living specimens from Luhuitou Reef, and yellow triangle represents fossil specimens from Meiji Reef)

Samples used in this study were collected from the coral reefs in the South China Sea (SCS, Figure 2). Living specimens were collected at Luhuitou fringing reef at Hainan Island in the northern SCS. They were taken by random sample using scuba diving at <10 m depth. Fossil specimens were selected from the drilled reef core Nanke‐1 (NK‐1) from Meiji Reef in the southern SCS. A total of 83 specimens were used for this study (Table S1).

### 2.2 Morphological identification

Living specimens were rinsed in freshwater using a high‐pressure water jet WaterPik to remove the soft tissue. The remaining skeletons were dried and then examined under a stereo dissecting microscope Olympus SZX7 and photographs taken with a usb microscope Anyty 3R‐MSUSB401. Each living specimen was identified to species level according to original taxonomic descriptions based on their skeleton macromorphological and micromorphological characters. The following references were used: Zou (2001), Wallace (1999), Veron (2000), Dai and Hong (2009). Taxonomic names were checked in the World Register of Marine Species (WoRMS, http://www.marinespecies.org/aphia.php?p=taxdetails&id=1363) and updated to reflect current taxonomic treatments.

### 2.3 Thin section preparation

Living coral specimens were first cut perpendicular to the growth axes of the corallites (transversal section) and then cut in half along corallites’ growth axis (longitudinal section). The reef core NK‐1 was first split in the middle and then cut into semicylinders at 10‐cm intervals. Fossil coral specimens were selected from semicylindrical slices and cut along and/or perpendicular to corallites’ growth axis as much as possible to obtain transversal/longitudinal sections. All sections were impregnated with a low viscosity epoxy resin and cut to 20–30 micrometer thickness.

### 2.4 Microstructure analysis

Thin sections were analyzed and photographed using an Olympus BX53‐P polarizing microscope, at magnifications ranging from 2× to 40×, equipped with a DP27‐CU noncooled color digital camera. In addition to many detailed microscopic photographs, whole panoramic photographs were combined with 2× microscopic photographs for each thin section in order to illustrate the overview of corallites and coenosteum. These were useful to confirm the presence of axial corallites and the arrangement of radial corallites for *Acropora* species.

### 2.5 Statistical analyses

To quantify the above descriptive characteristics for species delineations, we subjected measurements of the characters defined to cluster analysis and, for the potential development of a quantitative binary key, regression tree analysis. The Bray–Curtis similarity index and Ward method were used for cluster analysis. All species characters were used but some microstructural indicators played an important role in the regression tree analysis. Analyses were performed using the R software (R Foundation for Statistical Computing).

### 2.6 Terminology

**FIGURE 3:**
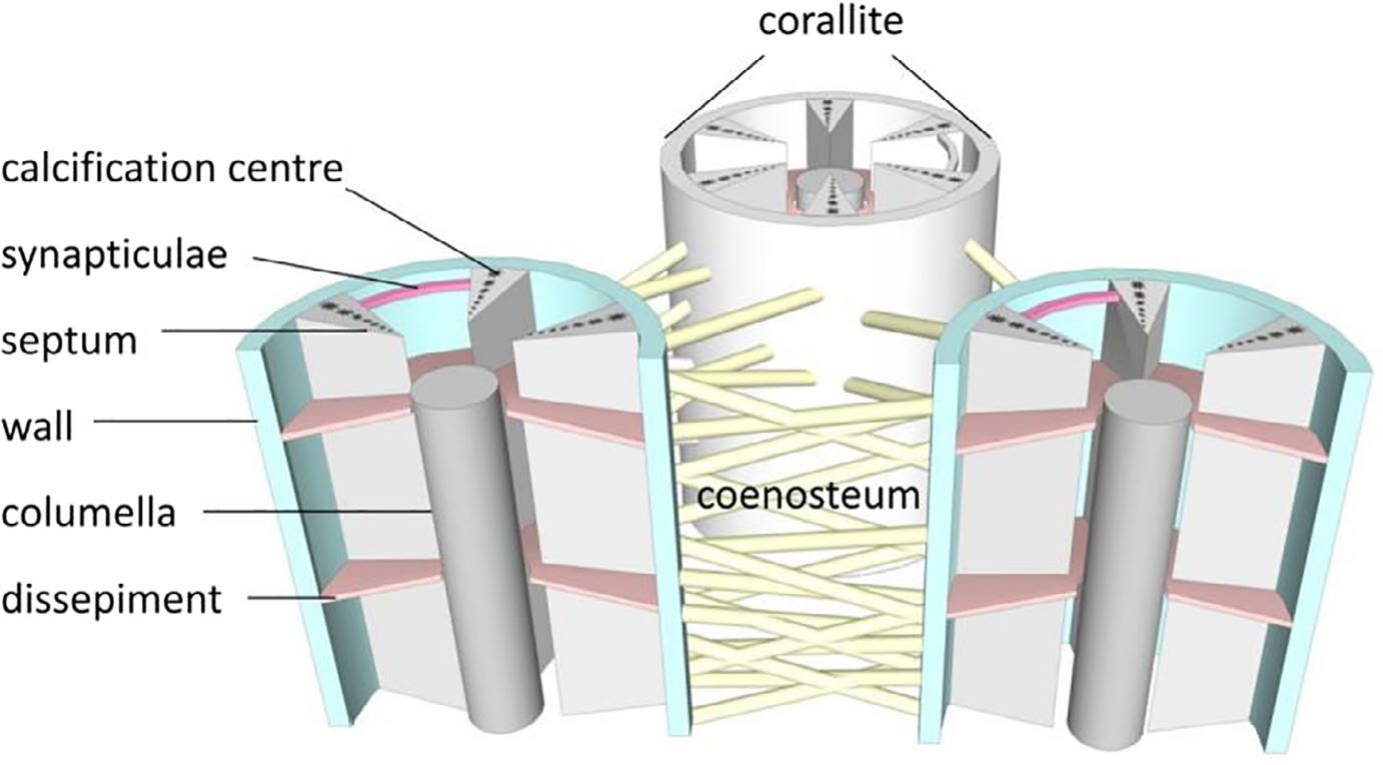
Graphic representation of some of the terms used to describe the coral structure

In the present paper, we define coral skeleton structures that can be seen in the thin sections with a microscope (Figure 3).

Corallite (‐s): skeleton of an individual polyp within a colony.
Coenosteum: skeleton between corallites.
Wall: skeletal structure uniting the outer edges of septa in a corallite.
Columella: skeletal elements present at the center of corallites.
Septum (‐a): vertical skeletal element radiating from the corallite wall toward the center. Septa are arranged in cycles. Primary septa are usually longer than secondary septa. In *Acropora*, one or two directive septa can often be distinguished and are distinctively longer than others.
Synapticulae: horizontal rods that extend between septa.
Dissepiment (‐s): small, horizontal plate inside or outside of a corallite, connecting vertical elements.
Calcification center: skeletal deposits as darkened areas at the center of trabeculae composed of tiny crystals, densely packed, randomly oriented, and embedded in an organic component.

## 3 RESULTS

### 3.1 Genus characteristics of *Acropora* and other five related genera

Based on living coral specimens and their skeletal characteristics illustrated from thin sections (transversal and longitudinal), ten characters were selected for genus identification (Table 1). All were characterized by small corallites, two septa cycles, and synapticular ring walls. More detailed microstructure characteristics were used to distinguish them in genus‐level analysis.

**TABLE 1:** The character analysis of the *Acropora* and other five related genus

Character	*Acropora*	*Isopora*	*Montipora*	*Astreopora*	*Pocillopora*	*Porites*
Corallite types	Dimorphic	Dimorphic	Monomorphic	Monomorphic	Monomorphic	Monomorphic
Axial corallites	Present	Present	Absent	Absent	Absent	Absent
Axial corallites number per branch	1	>1	0	0	0	0
Corallite diameter	Axial corallite outer diameter 1–3.9 mm, inner diameter 0.4–1.6 mm	Axial corallite outer diameter 2.5–4.5 mm, inner diameter 0.7–1.6 mm	0.4−1 mm	1–2.5 mm	0.7–1.5 mm	0.6–1.6 mm
Corallite wall	>1, porous	>3, porous	1, porous	1, solid	1, solid	1, porous
Septa	Two cycles, primary septa in axial corallite and directive septa in radial corallites well developed	Two cycles, primary septa in axial corallite and directive septa in radial corallites well developed	Two cycles, primary septa varies in length, directive septa occasionally meet, secondary septa short or absent	Two cycles, primary septa usually meet in the center and secondary septa short or absent	Septa degenerate to linear or discontinuous spikes, usually a smooth cavity without septa and columella	Two cycles, there are paliform tooth on the terminal of septa, and connected into a continuous or discontinuous ring
Columella	Absent	Absent	Absent/weakly represented	Weakly represented	Present	Present
Dissepiments	Absent	Absent	Absent	Present	Present	Present
Coenosteum	Reticular	Reticular	Reticular	Reticular	Solid	None
Calcification center	Line	Line	Cluster	Line	Line	Cluster

#### 3.1.1 Acropora

**FIGURE 4:**
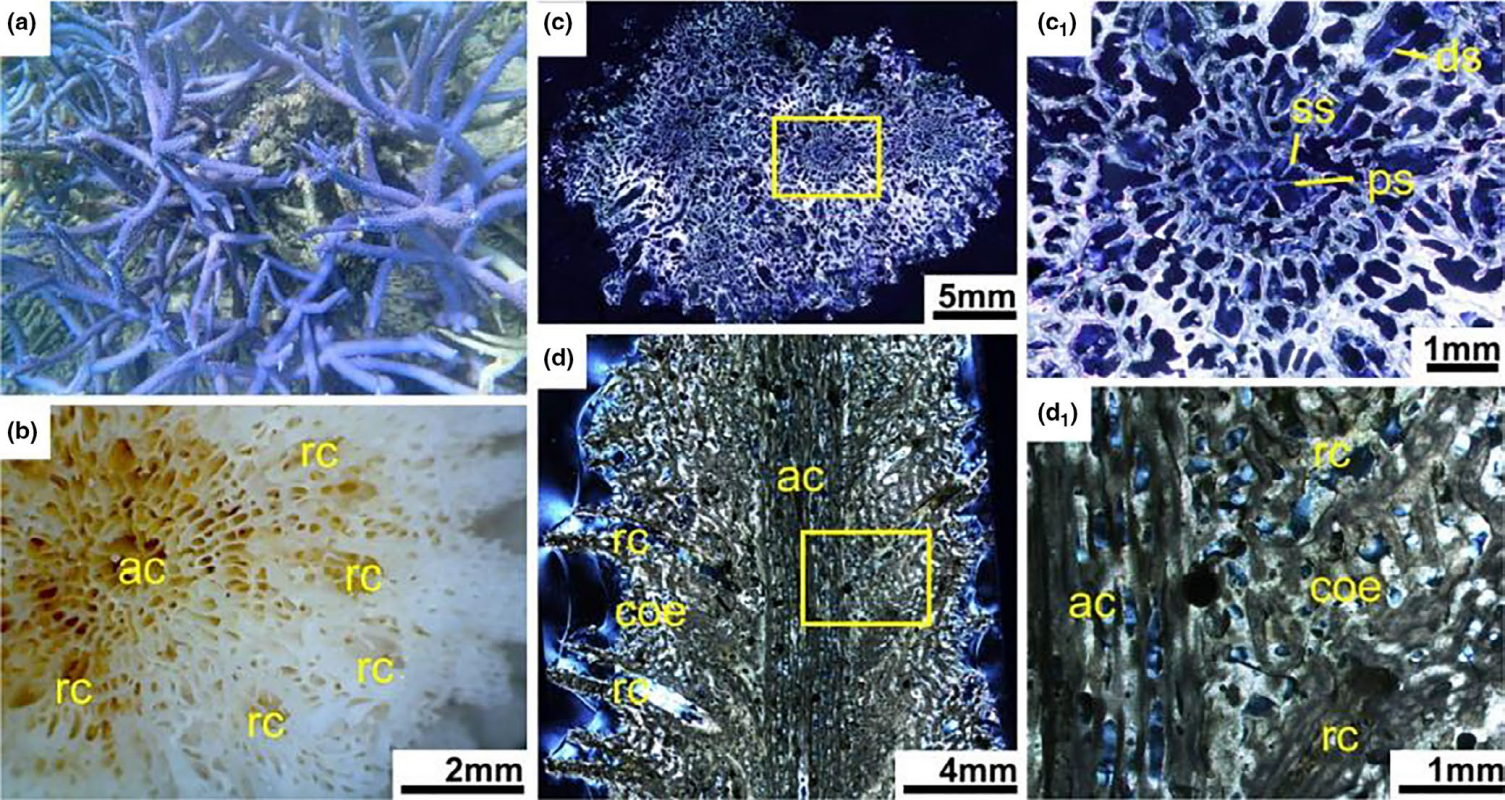
*Acropora formosa*. (a) Living colony underwater. (b) External skeleton macromorphology. (c) Internal skeleton microstructure of the transversal section. (c_1_) Detail of the transverse section. (d) Microstructure of the longitudinal section. (d_1_) Detail of the longitudinal section. ac, axial corallite; coe, coenosteum; ds, directive septa; ps, primary septa; rc, radial corallite; ss, secondary septa

Living *Acropora* colonies usually grow into various ramose shapes, including arborescent, hispidose, caespitose, corymbose, digitate, table or plate and rarely encrusting (Figure 4a). Each branch consists of a single axial corallite and numerous radial corallites (Figure 4b). Corallites are protuberant, with laminar or spinose septa, united by light reticulate coenenchyme, the surface of which is spinose or pseudocostate. There is no columella in the corallite.

In transversal thin section, the differentiation of axial corallites and radial corallites was distinctive (Figure 4c). The axial corallites were central and much larger than the surrounding radial corallites. Axial corallite outer diameter was about 1–3.9 mm, and its inner diameter ranged from 0.4 to 1.6 mm. They had mostly two cycles of six septa, primary septa usually well developed and secondary septal cycle sometimes absent, or some septa were missing. A pair of directive septa in the radial corallites were recognizable and more obvious than that in the axial corallites, indicating the bilateral plane of the corallite. Calcification centers were connected by medial lines within the septa. The walls of the axial and radial corallites were formed by the development of synapticular rings, their number varying from a single ring to several. The coenosteum between corallites was reticulate and very porous (Figure 4c1).

In longitudinal thin section, the dimorphism of corallites was also obvious, and axial corallites were central and made up the axis of branches (Figure 4d). Each branch was consisting of a single larger axial corallites and numerous smaller attendant radial corallites. There was no columella and dissepiments. The coenosteum was irregularly lengthwise furrowed (Figure 4d1).

#### 3.1.2 Isopora

**FIGURE 5:**
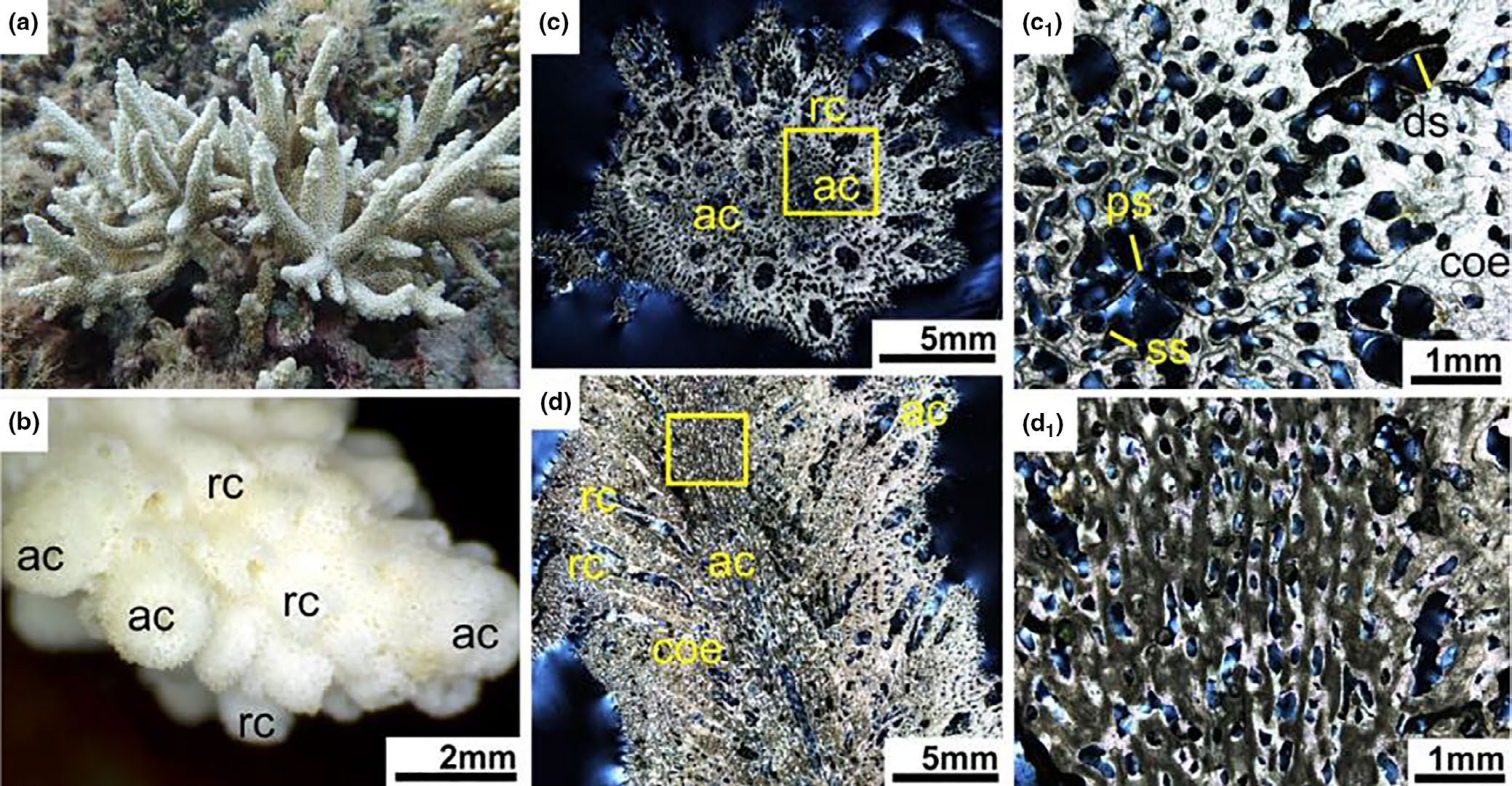
*Isopora brueggemanni* (a) Living colony underwater. (b) External skeleton macromorphology. (c) Internal skeleton microstructure of the transversal section. (c_1_) Detail of the transverse section. (d) Internal skeleton microstructure of the longitudinal section. (d_1_) Detail of the longitudinal section. ac, axial corallite; coe, coenosteum; ds, directive septa; ps, primary septa; rc, radial corallite; ss, secondary septa

Living *Isopora* colonies usually grow in ramose and encrusting shapes (Figure 5a). Different from branching *Acropora*, there exist multiple axial corallites, usually more than two. Coenosteum with elaborated meandroid spinules. No columella (Figure 5b).

In transversal thin section, adjacent axials were distinctive and much larger than ambient radials (Figure 5c) with outer diameters of 2.5–4.5 mm and inner diameters 0.7–1.6 mm. Two cycles of six septa, primary septa usually well developed and a complete or incomplete cycle of secondary septa. A distinctive pair of directive septa in the radials. Calcification centers connected by medial lines in the septa. More than three synapticular rings developed in the process of wall‐formation in the axials. The coenosteum between corallites reticulate and very porous (Figure 5c1).

In longitudinal thin section, dimorphism of corallites was also obvious and always more than one axial corallites were recorded, one of which formed the central axis of the main branch and was much larger than the numerous divergent smaller radials. There was no columella and dissepiments. The coenosteum was irregularly furrowed lengthwise (Figure 5d).

#### 3.1.3 Montipora

**FIGURE 6:**
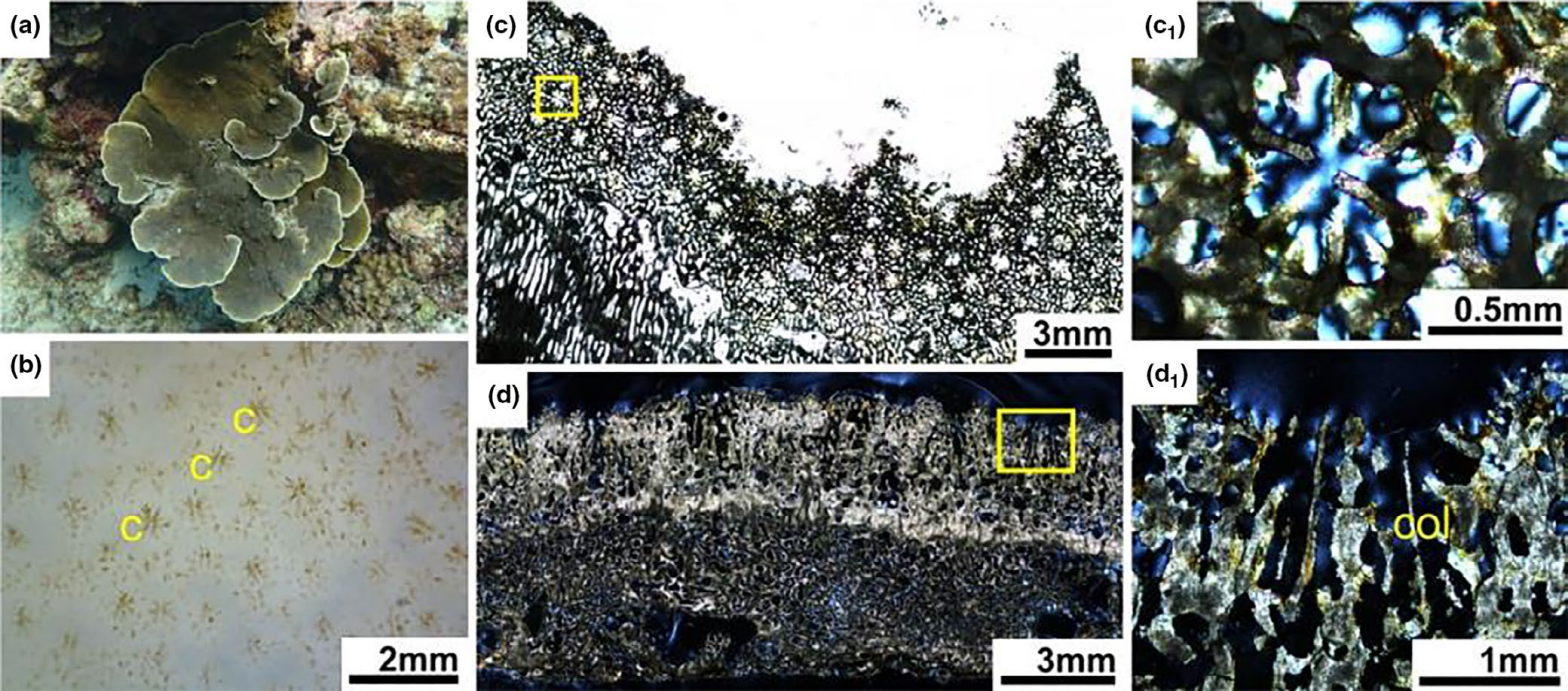
*Montipora monasteriata* (a) Living colony underwater. (b) External skeleton macromorphology. (c) Internal skeleton microstructure of the transversal section. (c_1_) Detail of the transverse section. (d) Internal skeleton microstructure of the longitudinal section. (d_1_) Detail of the longitudinal section. c, corallite; col, columellae

Living *Montipora* colonies can grow submassive, laminar, encrusting or branching (Figure 6a). Corallites are monomorphic and no axial corallites are developed. Corallite walls and the coenosteum are porous and may be elaborate (Figure 6b).

In transversal thin section, *Montipora* corallites were small (0.4‐1mm diameter). Septa were in two cycles, primary septa variable in length and secondary septa short or absent (Figure 6c). Septa formed by discrete clusters of calcification centers were different from calcification lines in the *Acropora*. Columellae absent or weakly developed. Corallite walls formed by a porous and discontinuous synapticular ring. The coenosteum between corallites porous with a regular mesh pattern (Figure 6c1). In longitudinal thin section, corallite walls porous. Columellae absent or feeble. Septa rudimentary, spinose. Coenenchyme reticulate, with sturdy vertical trabeculae and narrow horizontal connections. Surface spinose and spines often hirsute (Figure 6d).

#### 3.1.4 Astreopora

**FIGURE 7:**
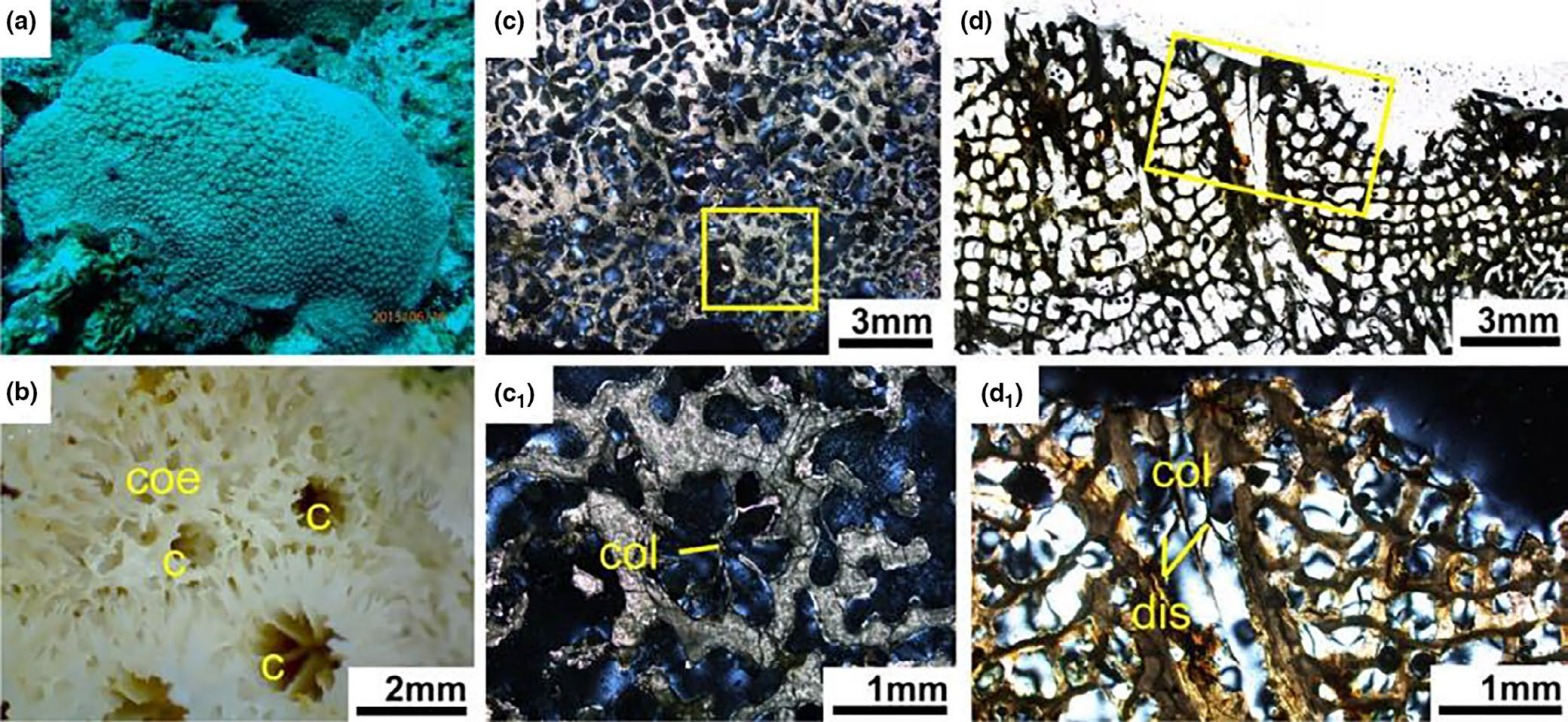
*Astreopora myriophthalma* (a) Living colony underwater. (b) External skeleton macromorphology. (c) Internal skeleton microstructure of the transversal section. (c_1_) Detail of the transverse section. (d) Internal skeleton microstructure of the longitudinal section. (d_1_) Detail of the longitudinal section. c, corallite; col, columellae; dis, dissepiment

Living *Astreopora* colonies are massive, laminar or encrusting. There are no axial corallites. Corallites immersed or conical (Figure 7a). In transversal thin section, *Astreopora* corallites ranging in diameter from 1–2.5 mm (Figure 7b). Septa in two irregular cycles, primary directive septa usually meet in the center and secondary septa short or absent. Calcification centers connected by medial lines in the septa. Corallite walls solid or slightly porous. Columellae present and obvious. The coenosteum porous (Figure 7c). In longitudinal thin section, columellae and dissepiments present. Coenenchyme reticulate, composed of trabeculae inclining outward from walls (Figure 7d).

#### 3.1.5 Pocillopora

**FIGURE 8:**
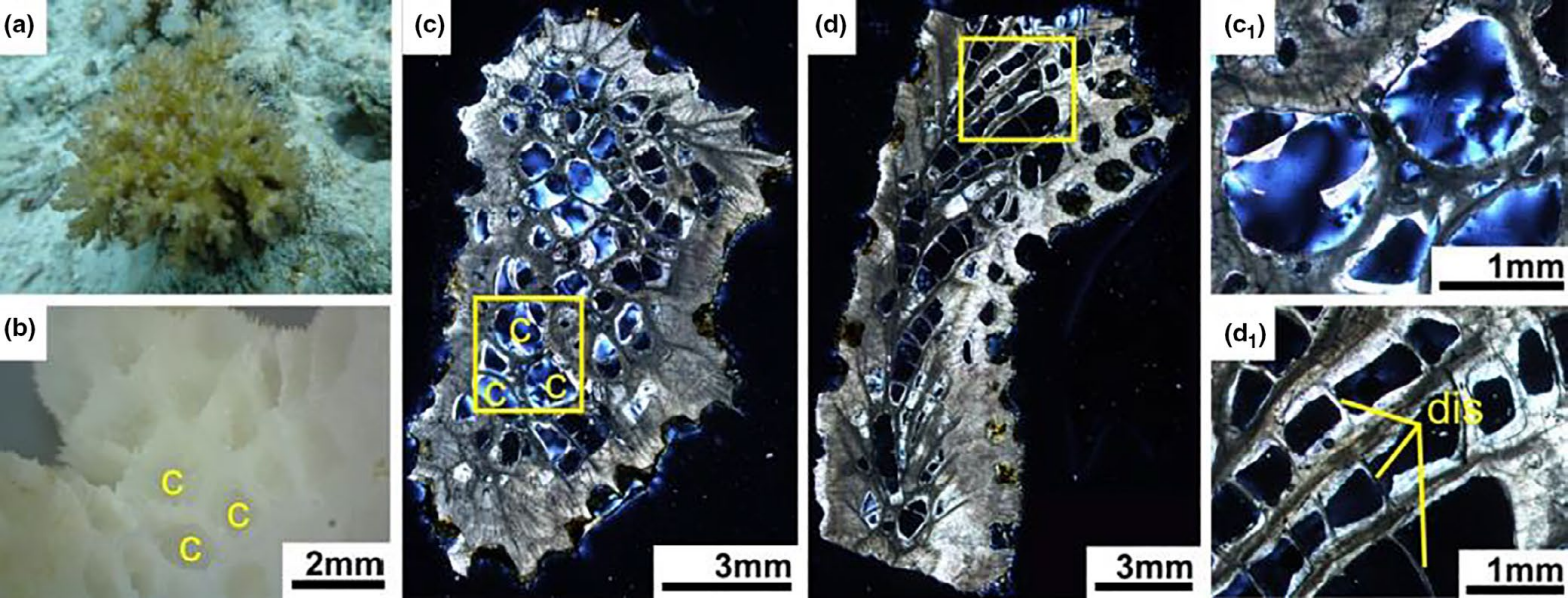
*Pocillopora damicornis* (a) Living colony underwater. (b) External skeleton macromorphology. (c) Internal skeleton microstructure of the transversal section. (c_1_) Detail characteristics of the transverse section. (d) Internal microstructure of the longitudinal section. (d_1_) Detail of the longitudinal section. c, corallite; dis, dissepiment

Living *Pocillopora* colonies are branching with branches either tending to be flattened or else fine and irregular (Figure 8a). No axial corallites. Corallites are small immersed. *Pocillopora* is readily distinguished from other genera by the presence of verrucae, which are skeletal protuberances that carry corallites (Figure 8b).

In transversal thin sections, diameter of *Pocillopora* corallites ranged 0.7–1.5 mm. Internal structure was significantly different from other genera. Septa were degenerate to linear or discontinuous spikes. Calices resembled smooth cavities without septa and columella. Corallite walls and coenosteum were solid (Figure 8c). From longitudinal thin section, dissepiments were obvious and created ladder shaped structures in the long cavity of corallites (Figure 8d).

#### 3.1.6 Porites

**FIGURE 9:**
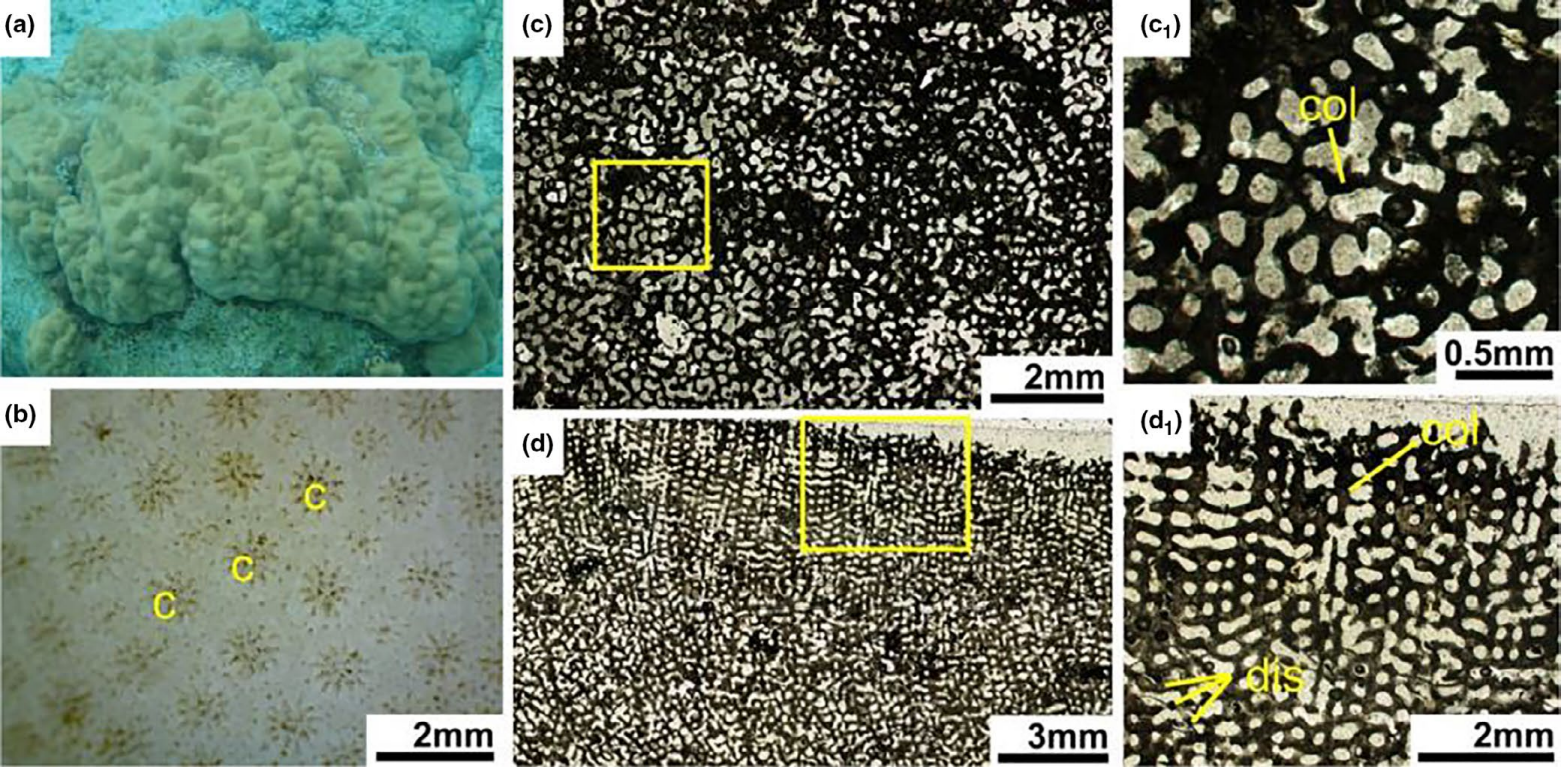
*Porites lutea*. (a) Living colony underwater. (b) External skeleton macromorphology. (c) Internal skeleton microstructure of the transversal section. (c_1_) Detail of the transverse section. (d) Internal microstructure of the longitudinal section. (d_1_) Detail of the longitudinal section. c, corallite; col, columellae; dis, dissepiment

Living *Porites* colonies are laminar, encrusting, massive or branching. Massive colonies can reach several meters across (Figure 9a). Axial corallites are absent. Corallites are small, immersed, circular or polygonal, crowded. Adjacent corallites often share a common wall and there is little or no coenosteum in between (Figure 9b).

In transversal thin section, *Porites* corallites diameter ranged 0.5–2.2 mm. Septa in two irregular cycles. Lateral septa often fuse to duplets, ventral septa frequently fused to triplets, sometimes with fused sides, the dorsal septum unfused and shorter than the others. Septa formed by discrete clusters of calcification centers. Pali present, variable development in different species, usually 4–8 in number. Columellae present. The wall formed by a synapticular ring (Figure 9c). In longitudinal thin sections, columellae and dissepiments were present (Figure 9d).

### 3.2 Interspecific characteristics within the genus of *Acropora*

For the analysis of relationships within genus *Acropora*, eighteen skeletal characters were summarized (Table 2). Skeletal characters included those relating to axial corallites, radial corallites and coenosteum. A total of ten *Acropora* species common at coral reefs in the SCS were analyzed, and their character states are listed in Table S2.

**TABLE 2:** Characters revealed from thin section and character states used in the identification of the *Acropora* species

Character No.	Character	States	Coding
1	Axial corallites outer diameter	Small (<2 mm)	0
		Medium (2−3 mm)	1
		Large (>3 mm)	2
2	Axial corallites inner diameter	Small (<1 mm)	0
		Medium (1–1.5 mm)	1
		Large (>1.5 mm)	2
3	Axial corallites synapticular rings	2	0
		3	1
		>3	2
4	Axial corallites synapticular cavity filling	No	0
		Yes	1
5	Axial corallites primary septa length	Short (<2/3R)	0
		Medium (2/3R−3/4R)	1
		Long (>3/4R)	2
6	Axial corallites secondary septa cycle	Incomplete	0
		Complete	1
7	Axial corallites septa connectivity	Septa not connect	0
		Some septa connected	1
8	Axial corallites septa top swelling	No swelling	0
		Swelling	1
9	Axial corallites septa calcification lines width	Thin	0
		Thick	1
10	Axial corallites septa calcification lines curving	Straight	0
		Curve	1
11	Radial corallites synapticular rings	2	0
		3	1
		>3	2
12	Radial corallites primary septa cycle	Incomplete	0
		Complete	1
13	Radial corallites primary septa length	Short (<1/2R)	0
		Medium (1/2R−2/3R)	1
		Long (>2/3R)	2
14	Radial corallites directive septa directivity	Outside septa better developed	0
		Inside septa better developed	1
		Equalization	2
15	Coenosteum arrangement	Less regular	0
		Regular	1
16	Coenosteum mesh size	Small	0
		Large	1
17	Coenosteum lateral binding	No	0
		Yes	1
18	Coenosteum marginal palisading arrangement	No	0
		Yes	1

#### 3.2.1 Axial corallites

Axial corallites were the most conspicuous skeletal units in the genus *Acropora*. Its microstructure characteristics were well recorded in thin section. They were central and surrounded by numerous radial corallites in transversal thin sections (Figure 4c). Axials were continuous along the entire branch in longitudinal thin sections (Figure 4d). Axials can be categorized as small, medium, and large in terms of outer and inner diameter. Axial corallites of *A*. *robusta* were very large with outer and inner diameters up to 4.5 mm and 2.8 mm, respectively, while small axial corallites of *A*. *cerealis* had mean outer and inner diameter of 1.5 mm and 0.8 mm, respectively.

The walls of axial corallites consisted of porous synapticular rings. The number of such rings varied among species, from two to more than three (Table 2). In addition to number of rings, infilling of the cavities of synapticular rings by an aragonite stereome without calcification center was a distinctive characteristic. Synapticular ring cavities of *A. valida*, *A. hyacinthus,* and *A*. *florida* were mostly filled (Table S2).

Axial corallites had two cycles of six septa. Primary septa usually complete and well developed. The length of primary septa varied among species. Longer septa could reach up to 3/4 of the corallite's radius (e.g., *A*. *millepora, A. robusta, A. abrotanoides*), shorter septa close to 2/3R (e.g., *A*. *pulchra, A. cerealis*). Secondary septa cycle mostly poorly developed. Twelve septa were present in only three species (*A*. *robusta, A. muricata, A. millepora*). The shape of septa in the thin section allowed species differentiation. The connection of secondary septa to neighboring primary septa and swollen ending of septa were common in *A*. *robusta* and *A. millepora*. The terminus of septa in *A. abrotanoides* and *A. muricata* were slightly swollen but did not cause connection between the septa (Table S2).

Calcification centers were in closely arrangement and connected to medial lines in the septa of axial corallites in the genus of *Acropora*. The calcification lines usually were thick and straight in most *Acropora* species, but thinner and irregular calcification lines with different curvatures were found in *A. abrotanoides*, *A*. *cerealis,* and *A. muricata* (Table S2).

#### 3.2.2 Radial corallites

Radial corallites bud from the central axial corallite. Diameter size and arrangement were highly variable among and within species. Walls of radials consisted of porous synapticular rings and could be used to distinguish species. The number of rings usually was equivalent to that in the axials, except in *A. millepora* and *A. pulchra*, which had three synapticular rings in the axials and only two rings in radials (Table S2).

Radial corallites could had two cycles of six septa. The primary septa cycle was usually undeveloped in the radials. The number of primary septa was incomplete in most cases, all six primary septa present in the minority of species (*A*. *valida*, *A*. *tenuis*, *A. florida,* and *A*. *abrotanoides*). If primary septa were present in the radials, they were shorter than in the axials. The length of primary septa varied in the radials of different species. Primary septa were up to 2/3R in three species and less than 1/2R in another three (Table S2). A pair of directive septa was recognizable, indicating the bilateral plane of the radials. The dominance in the length of inner side or outer side (close to or away from the central axial corallites) of directive septa would be used for identifying different *Acropora* species. For example, inner side of directive septa was developed better than outer side in the species of *A*. *abrotanoides*, *A*. *hyacinthus,* and *A*. *muricata*, with the opposite in *A*. *cerealis*, *A*. *florida*, *A*. *millepora,* and *A. robusta* (Table S2).

#### 3.2.3 Coenosteum

The coenosteum of *Acropora* was reticular and appeared as a mesh with different pore size. Arrangement mode, mesh size, lateral binding, and marginal palisading varied among species (Table 2). The coenosteum of *A*. *hyacinthus*, *A*. *muricata*, *A*. *millepora,* and *A*. *pulchra* had a relatively regular reticular arrangement compared to the other six species. Mesh size was largest in *A*. *muricata*, *A*. *abrotanoides*, and *A*. *pulchra*. Lateral binding of coenosteum differed and was significant in *A*. *cerealis* but only slight in *A*. *abrotanoides*. Palisade structures existed in the coenosteum of marginal of branches in *A*. *hyacinthus*, *A*. *tenuis*, *A*. *cerealis,* and *A*. *florida* (Table S2).

#### 3.2.4 Quantification of morphological differences

**FIGURE 10:**
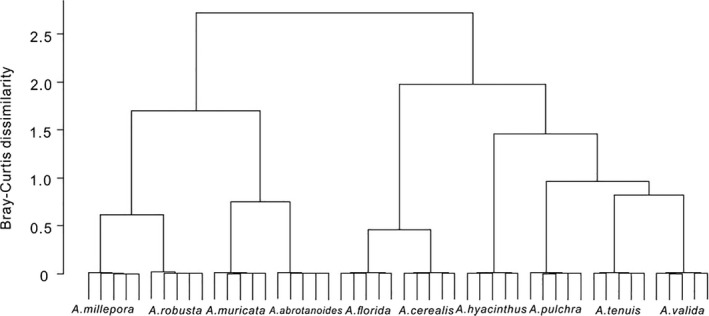
Cluster analysis (Ward method of linkage) of *Acropora* species based on the skeleton characters from thin sections. Species fell into distinct and well‐defined clusters supporting the validity of the chosen characters

**FIGURE 11:**
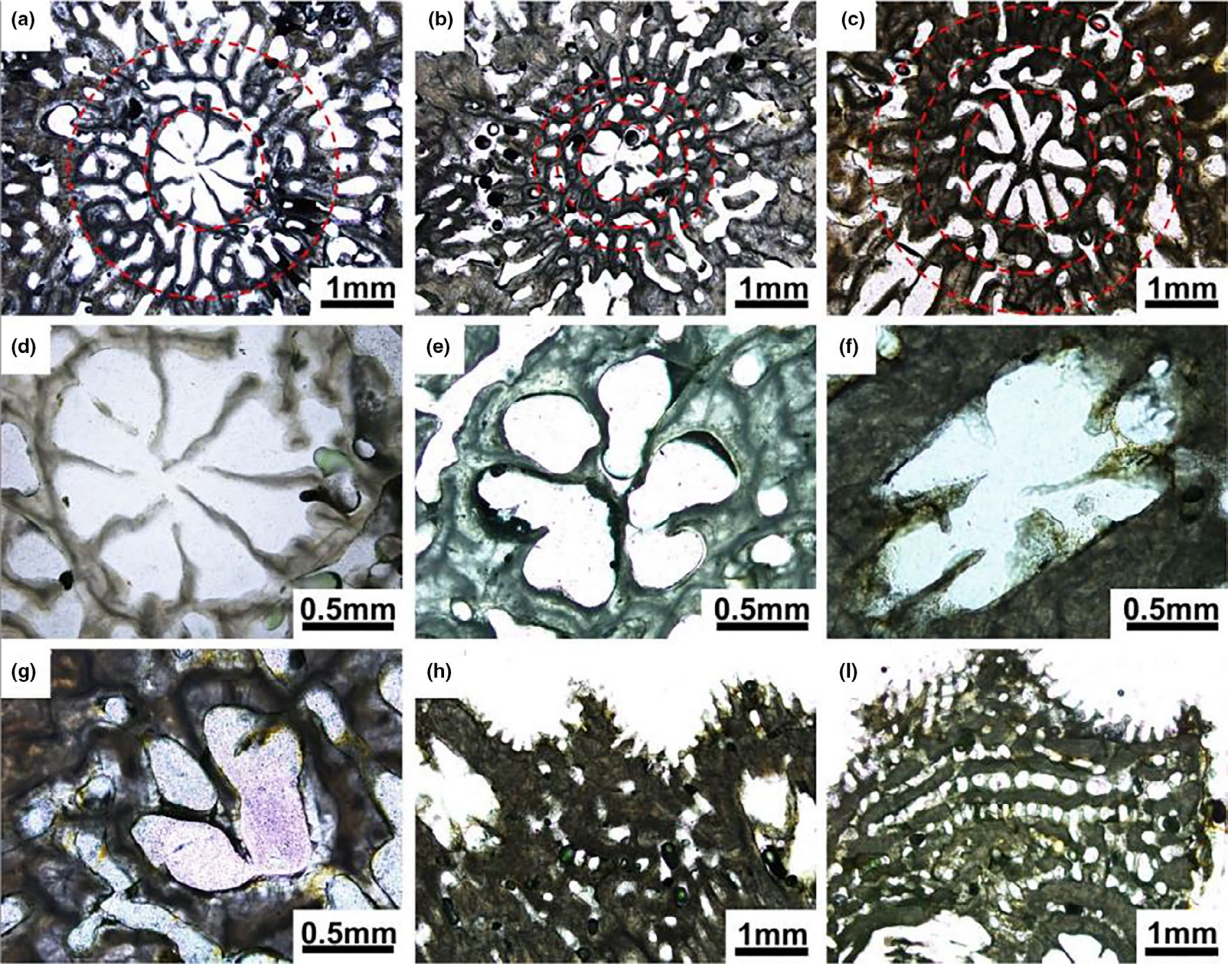
The most important characters for differentiation of *Acropora* species: (1) axial corallites outer diameter, indicating large size (a: *A*. *abrotanoides*) or small size (b: *A*. *cerealis*); (2) axial corallites synapticular rings, indicating the structure of wall with two synapticular rings (a: *A*. *abrotanoides*) or three synapticular rings (c: *A*. *millepora*); (3) axial corallites primary septa length, indicating longer septa (d: *A*. *abrotanoides*) or shorter septa (e: *A*. *florida*); (4) radial corallites primary septa cycle, indicating complete developed six primary septa (f: *A*. *abrotanoides*) or some primary septa absent (g: *A*. *millepora*); and (5) coenosteum marginal palisading arrangement, indicating the coenosteum without (h: *A*. *abrotanoides*) or with palisade structure in the margin (i: *A* .*tenuis*)

**FIGURE 12:**
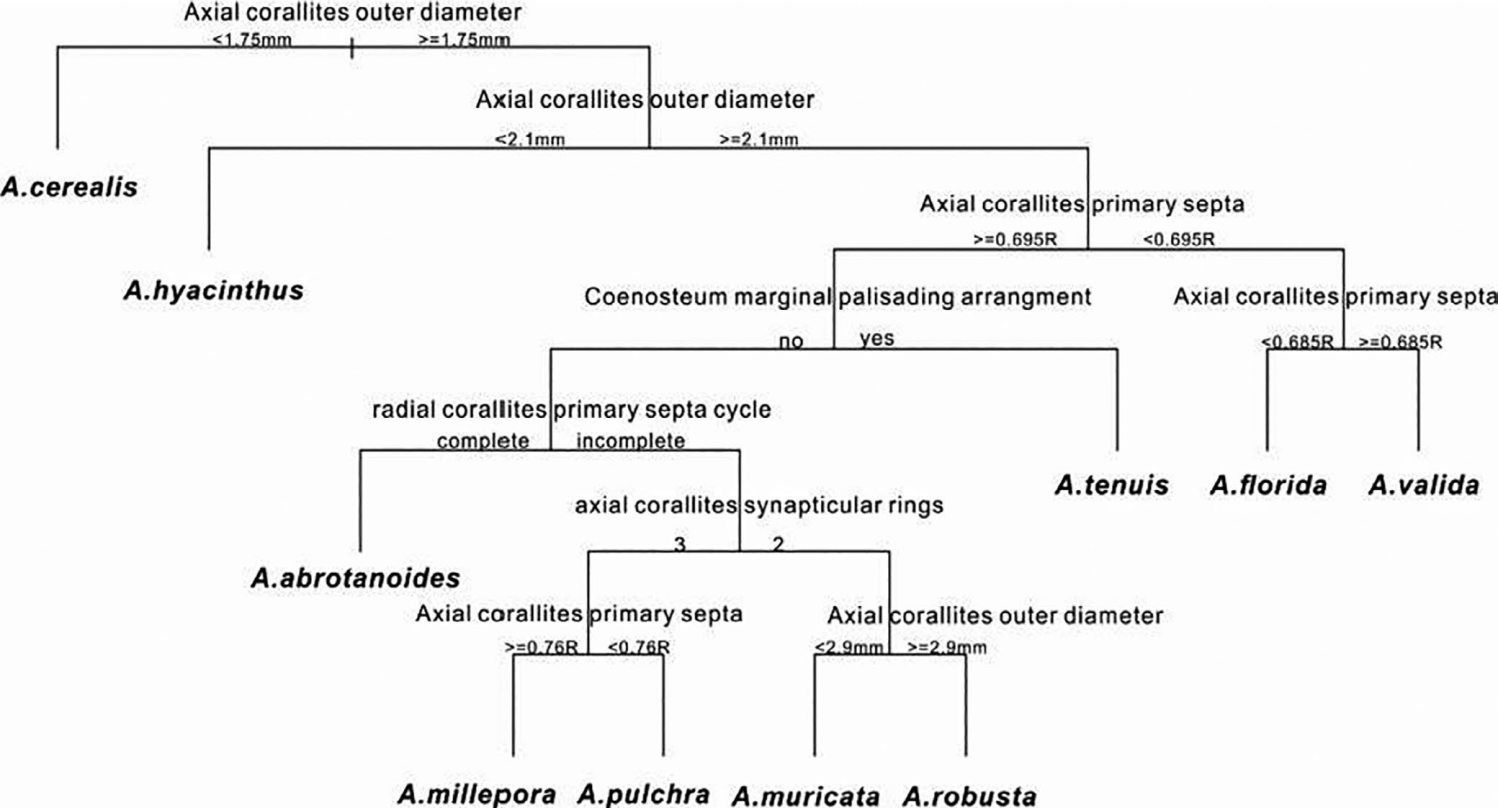
Regression tree analysis of *Acropora* species as help to developing a taxonomic tree. The analysis identifies the most important skeleton characters from thin sections that serve to separate species

Cluster analysis suggested that the measured variables were indeed appropriate for the differentiation of species, since the clusters grouped specimens of the same species (Figure 10). The regression tree analysis suggested that the following characters were the most important for differentiation of species: axial corallite outer diameter, axial corallite synapticular rings, axial corallite primary septa, radial corallite primary septa, coenosteum marginal palisading arrangement (Figure 11). The regression tree separates species with axial corallites < 2.1 mm diameter (*A. cereali*s and *A. hyacinthus*) from the rest, which again separate into species with shorter primary septa of axial corallites (<0.5 mm, *A. valida* and *A. florida*) from the rest. The marginal palisading arrangement then separates *A. tenuis*. *A*. *abrotanoides* differs from all others for its complete developed six primary septa of radial corallites. *A. millepora* and *A*. *pulchra* are separated for three synapticular rings from *A*. *muricata* and *A*. *robusta* with two synapticular rings (Figure 12).

## 4 DISCUSSION

### 4.1 Application of genus identification to the reef core NK‐1 specimens

The identification of fossil reef corals is important for the investigation of ancient reefs and the change of coral communities along the geological time (Hongo & Kayanne, 2011; Humblet et al., 2015), especially for the paleoecological responses of coral community to paleoenvironmental changes (Pandolfi, 1996, 2011) and evolutionary studies looking at speciation and extinction events (Budd, 2000). The taxonomic identification of fossil reef corals is often limited by their poor preservation of external skeleton characteristics. The macromorphological characteristics of the surfaces of coral skeletons are the main foundation for the taxonomy of modern reef corals (Veron, 2000). Less information is available regarding the link between the internal skeleton structure of scleractinian corals and their external morphology (Budd et al., 2012; Huang et al., 2016). The internal and surficial characteristics of coral skeletons were recorded in thin sections, and the connection between fossil reef corals from the drilled cores and modern reef corals from underwater field survey was established. Only ten microstructural characteristics of these six genera (Table 1) allowed fifteen fossil coral species to be easily recognized and identified on genus level.

**FIGURE 13:**
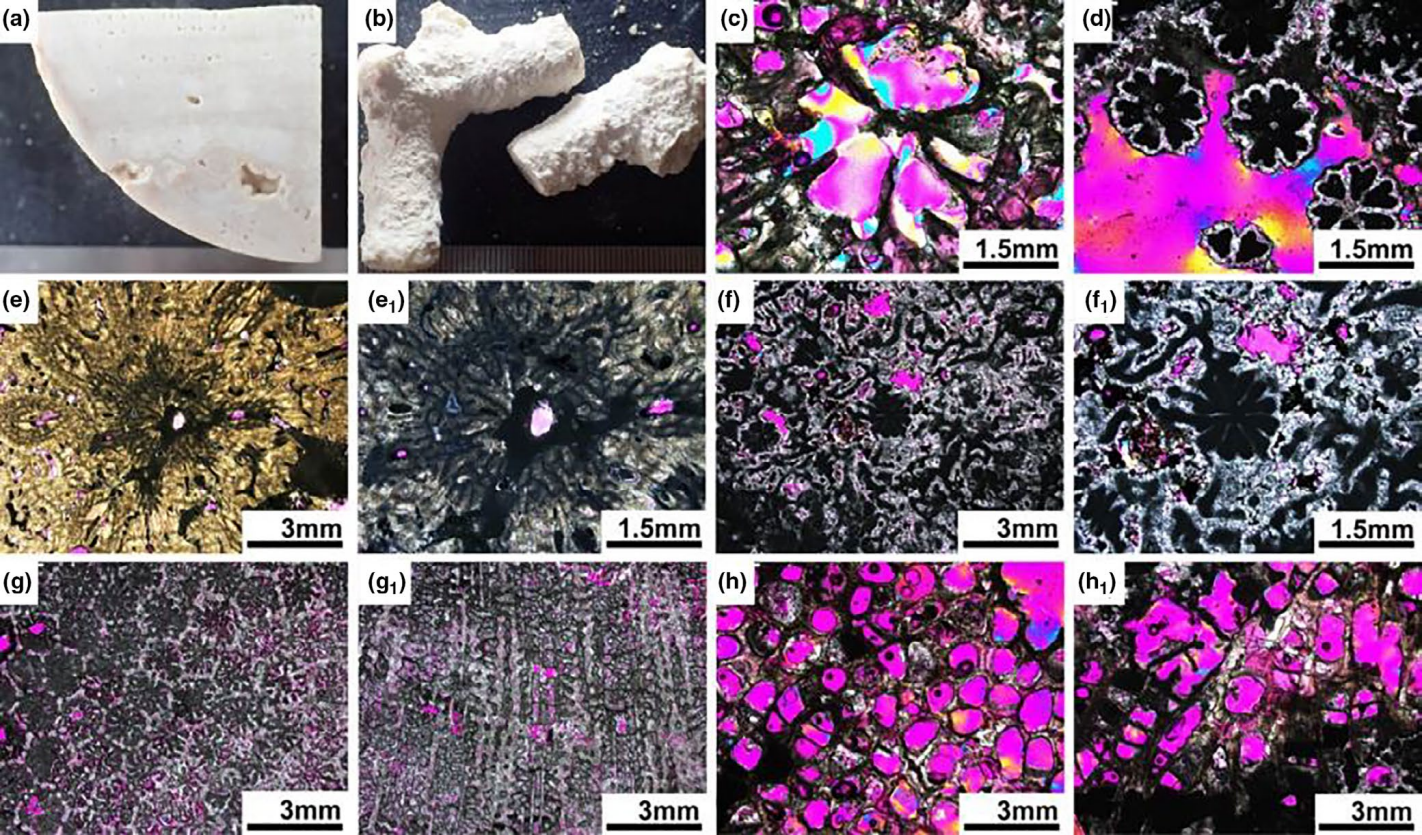
Fossil specimens from core Nanke‐1 at Meiji Reef in the South China Sea. (a) Core slice from dense component of drilled reef core. (b) Hand specimens from loose component of drilled reef core. (c) The sample of NK‐1‐0234, the transverse section of an *Astreopora* corallite. (d) The sample of NK‐1‐2652, the transverse section of *Montipora* corallites. (e) The sample of NK‐1‐0062, the transverse section of *Acropora* corallites. (e_1_) Details of axial corallite and radial corallites of NK‐1‐0062. (f) The sample of NK‐1‐3416, the transverse section of *Isopora* corallites. (f_1_) Details of axial corallite and radial corallites of NK‐1‐3416. (g) The sample of NK‐1‐5833, the transverse section of *Porites* corallites. (g_1_) The sample of NK‐1‐6267, the longitudinal section of *Porites* corallites. (h) The sample of NK‐1‐0239, the transverse section of *Pocillopora* corallites. (h_1_) The sample of NK‐1‐0239, the longitudinal section of *Pocillopora* corallites

*Acropora* and *Isopora* were easily distinguished by a unique form of dimorphic corallites: axial and radial. Axial corallites are cylindrical and may reach several centimeters in length, while radial corallites occur in a variety of shapes and are never more than a few millimeters long. *Isopora* was proposed as a subgenus (Veron & Wallace, 1984; Wallace, 1999) and was elevated to genus recently based on morphological and genetic analyses (Fukami et al., 2000; Wallace et al., 2007). *Acropora* is currently defined on the basis of having branches formed only around a single axial corallites and broadcast‐spawning for external fertilization. This differentiates them from *Isopora* which possess more than one axial corallite and brood planula larvae. Although *I*. *brueggemanni* only sometimes showed more than a single axial corallites and frequent had distinct single axial corallites, it was distinguished from *Acropora* by having more than three synapticular rings and well developed primary septa in radial corallites—characteristics clearly seen in thin sections (Figures 5c and 13).

Besides the differentiation of axial and radial corallites, the distinctive structure of the corallite wall of *Acropora* set it apart from the genera *Montipora* and *Astreopora* in the same family Acroporidae. The corallites wall of *Montipora* were also porous but had only one synapticular ring, while *Astreopora* possessed a solid corallite wall. The columella was always absent in *Acropora*, but in *Montipora* could be either absent or weakly represented, while in *Astreopora* it was usually present (Figure 13). *Montipora* and *Astreopora* also showed clear differences in the arrangement of calcification centers in the septa. They clustered in the former and were arranged in medial lines in the latter (Figures 4, 5 and 9).

*Pocillopora* and *Porites* were used for comparison with *Acropora*, because these two genera were regarded as containing plesiomorphic characters shared with the family Acroporidae, such as small corallites, two septal cycles and growth forms (branching *Pocillopora* like *Acropora* and submassive and encrusting of *Porites* like *Montipora* and *Astreopora*). Fossil *Pocillopora* was easily distinguished in the transversal thin section by smooth cavities of corallites with rudimentary septa and columella. In the longitudinal thin sections by ladder‐like pattern of tabulate dissepiments and solid corallites wall and coenosteum (Figure 13). Fossil *Porites* was also clearly identified relying by a ring of pali around the columella in the transversal thin section. In the longitudinal thin sections, the columella and regular corallite arrangement was typical (Figure 13).

### 4.2 Prospect of fossil *Acropora* species identification for paleoecology and modern conservation

*Acropora* is distinguished by its exclusively axial branching mode, and differentiation of axial and radial corallites, with associated coenosteal differentiation, such that 20 species groups have been recognized (Wallace, 1999). Axial corallites were obvious in longitudinal and transversal thin sections. Eighteen skeleton characteristics from thin sections allowed for reliable species identification. In view of the fragility of *Acropora* branches and the influence of biogeochemistry, burial diagenesis and dissolution on the quality of reef cores, many skeletons of fossil *Acropora* tend to be incomplete or even replaced by other rock constituents (Humblet et al., 2015). But using these eighteen skeleton characteristics, the axial corallites size and structure (including its diameter, synapticular rings and septa), the septa of radial corallites and the arrangement of coenosteum most fossil *Acropora* species, unless very badly preserved, should be identifiable. For example, both fossil *A*. *hyacinthus* and *A*. *tenuis* exhibited two synapticular rings in the corallite walls, lateral binding coenosteum and marginal palisading arrangement. Specimens could be identified to species level by relying on *A. hyacinthus* having smaller axial corallites and well developed directive septa. Both fossil *A*. *cerealis* and *A*. *florida* had three synapticular rings and well developed outside directive septa. They could be separated by larger size axial corallites with thick and longer septa in *A. florida*.

*Acropora* is important in the modern coral communities due to high species diversity (135 extant species) and rapid growth (up to more than 10cm/year) and it also played a major role forming fossil reef framework (Hongo & Kayanne, 2011; Montaggioni, 2005). In the Caribbean, Holocene reefs were dominated by *A. palmata* and *A. cervicornis* (Aronson et al., 2005; Gischler & Hudson, 2004). In the Indo‐Pacific, the distribution of *Acropora* species in the present ocean has been intensely studied (Veron, 2000; Wallace, 1999), but reconstructions of reef growth history are usually based on data derived from growth forms and combinations of certain species. For example, *Acropora* became a dominant reef builder during the Middle Pleistocene on the Great Barrier Reef (GBR), robust branching *Acropora* gr. *humilis* and *Acropora* gr. *robusta*, and arborescent *Acropora* gr. *formosa* were found in different positions of cores and reef stages (Humblet & Webster, 2017). In addition, the dominant corals in Mauritius were in the *A*. *robusta/abrotanoides* complex 6,000 years ago (Hongo & Montaggioni, 2015), and the Miocene of East Kalimantan was dominated by the species in the *horrida*, *humilis,* and *elegans* groups (Santodomingo et al., 2016). Species‐level identification remains to be performed in many areas, and the distribution patterns of species during past reef formation remain poorly understood.

The species‐level records from fossil corals could show their ecological adaptability to various environmental change in the geological time (Edmunds et al., 2014; Santodomingo et al., 2016). The repeated occurrence of similar coral assemblages characterized by robust branching corals (*Acropora* gr *humilis* and *Acropora* gr *robusta*) in cores indicates that the Great Barrier Reef has been able to reestablish itself over the last 500 ka, despite major environmental fluctuations in sea level and perhaps temperature (Webster & Davies, 2003). *A*. *cervicornis* in the Caribbean Holocene reefs flourished during a 4000‐yr period and survived large‐scale climate and environmental changes that included high temperatures, variable salinity, hurricanes, and rapid sea‐level rise displayed remarkable resilience (Greer et al., 2009; Wapnick et al., 2004). *Acropora* even was one of the most dominant Scleractinia taxa from paleoecological inventory of the nearshore turbid‐zone reef complex on the central GBR, mainly including arborescent species, for example, *A. muricata and A. pulchra* (Johnson et al., 2017; Perry et al., 2008; Ryan et al., 2016).

## 5 CONCLUSION

Skeleton characteristics from thin section, which represent a link between the internal skeleton structure and external morphology, allowed definition of ten characteristics that allowed to distinguish *Acropora* and five related genera at the genus level. Eighteen characters (ten of axial corallites, four of radial corallites, and four of coenosteum) allowed *Acropora* species classification. Axial corallites size and structure (diameter, synapticular rings, and septa), the septa of radial corallites, and the arrangement of coenosteum were important for fossil *Acropora* species identification.

## CONFLICT OF INTEREST

The authors declare that they have no conflict of interest.

## AUTHOR CONTRIBUTION

**Meixia Zhao:** Conceptualization (lead); Data curation (equal); Formal analysis (equal); Methodology (equal); Project administration (lead); Software (equal); Writing‐original draft (equal); Writing‐review & editing (equal). **Haiyang Zhang:** Data curation (equal); Formal analysis (equal); Software (equal); Writing‐original draft (equal). **Yu Zhong:** Data curation (equal); Formal analysis (equal); Software (equal); Writing‐original draft (equal). **Xiaofeng Xu:** Data curation (equal); Formal analysis (equal); Software (equal); Writing‐original draft (equal). **Hongqiang Yan:** Resources (equal). **Gang Li:** Resources (equal). **Wen Yan:** Funding acquisition (equal); Methodology (equal); Project administration (equal); Resources (equal).

## Supporting information

Tables S1‐S2

## Data Availability

The data are available in the Dryad Data Repository (https://doi.org/10.5061/dryad.wh70rxwmp).
