# Supplementary material for: Microstructural characteristics of the stony coral genus Acropora useful to coral reef paleoecology and modern conservation
Source: Ecol Evol. 2021 Mar 9;11(7):3093–109. doi: 10.1002/ece3.7247 (PMC8019043; doi:10.1002/ece3.7247)
Supplement: Supplementary file 1 — Tables S1‐S2 [file ECE3-11-3093-s001.docx]

**Appendix**

Table S1 The living and fossil coral specimens were collected and analyzed in this research.

| No. | Sample | *Species* | Type | Source |
| --- | --- | --- | --- | --- |
| 1 | N1-1-003 | *Montipora peltiformis* | living | Luhuitou |
| 2 | N1-1-004 | *Montipora peltiformis* | living | Luhuitou |
| 3 | N1-1-011 | *Montipora efflorescens* | living | Luhuitou |
| 4 | N1-1-012 | *Montipora efflorescens* | living | Luhuitou |
| 5 | N1-1-013 | *Acropora muricata* | living | Luhuitou |
| 6 | N1-1-014 | *Acropora muricata* | living | Luhuitou |
| 7 | N1-1-019 | *Porites lobata* | living | Luhuitou |
| 8 | N1-1-020 | *Porites lobata* | living | Luhuitou |
| 9 | N1-1-029 | *Porites lutea* | living | Luhuitou |
| 10 | N1-1-030 | *Porites lutea* | living | Luhuitou |
| 11 | N1-1-035 | *Acropora cerealis* | living | Luhuitou |
| 12 | N1-1-036 | *Acropora cerealis* | living | Luhuitou |
| 13 | N1-1-043 | *Porites solida* | living | Luhuitou |
| 14 | N1-1-044 | *Porites solida* | living | Luhuitou |
| 15 | N1-1-059 | *Astreopora myriophthalma* | living | Luhuitou |
| 16 | N1-1-060 | *Astreopora myriophthalma* | living | Luhuitou |
| 17 | N1-1-067 | *Pocillopora damicornis* | living | Luhuitou |
| 18 | N1-1-068 | *Pocillopora damicornis* | living | Luhuitou |
| 19 | N2-1(1)-003 | *Acropora cerealis* | living | Luhuitou |
| 20 | N2-1(1)-004 | *Acropora cerealis* | living | Luhuitou |
| 21 | N2-1(1)-011 | *Astreopora myriophthalma* | living | Luhuitou |
| 22 | N2-1(1)-012 | *Astreopora myriophthalma* | living | Luhuitou |
| 23 | N2-1(1)-037 | *Montipora peltiformis* | living | Luhuitou |
| 24 | N2-1(1)-038 | *Montipora peltiformis* | living | Luhuitou |
| 25 | N2-1(1)-057 | *Porites lutea* | living | Luhuitou |
| 26 | N2-1(1)-058 | *Porites lutea* | living | Luhuitou |
| 27 | N2-1(1)-059 | *Porites solida* | living | Luhuitou |
| 28 | N2-1(2)-035 | *Montipora efflorescens* | living | Luhuitou |
| 29 | N2-1(2)-036 | *Montipora efflorescens* | living | Luhuitou |
| 30 | N2-1(2)-053 | *Pocillopora damicornis* | living | Luhuitou |
| 31 | N2-1(2)-054 | *Pocillopora damicornis* | living | Luhuitou |
| 32 | N2-1(2)-055 | *Porites lobata* | living | Luhuitou |
| 33 | N2-1(2)-056 | *Porites lobata* | living | Luhuitou |
| 34 | N2-1(2)-064 | *Acropora hyacinthus* | living | Luhuitou |
| 35 | N2-1(2)-065 | *Acropora hyacinthus* | living | Luhuitou |
| 36 | N2-1(2)-066 | *Acropora robusta* | living | Luhuitou |
| 37 | N2-1(2)-067 | *Acropora robusta* | living | Luhuitou |
| 38 | N2-1(2)-078 | *Acropora abrotanoides* | living | Luhuitou |
| 39 | N2-1(2)-079 | *Acropora abrotanoides* | living | Luhuitou |
| 40 | N2-1(2)-080 | *Acropora florida* | living | Luhuitou |
| 41 | N2-1(2)-080 | *Acropora florida* | living | Luhuitou |
| 42 | N2-1(2)-081 | *Acropora florida* | living | Luhuitou |
| 43 | N2-1(2)-098 | *Montipora foliosa* | living | Luhuitou |
| 44 | N2-1(2)-099 | *Montipora foliosa* | living | Luhuitou |
| 45 | N2-1(2)-100 | *Montipora monasteriata* | living | Luhuitou |
| 46 | N2-2(1)-012 | *Montipora tuberculosa* | living | Luhuitou |
| 47 | N2-2(1)-013 | *Montipora tuberculosa* | living | Luhuitou |
| 48 | N2-2(1)-014 | *Pocillopora verrucosa* | living | Luhuitou |
| 49 | N2-2(1)-015 | *Pocillopora verrucosa* | living | Luhuitou |
| 50 | N2-2(1)-028 | *Montipora hispida* | living | Luhuitou |
| 51 | N2-2(1)-029 | *Montipora hispida* | living | Luhuitou |
| 52 | N2-2(1)-034 | *Porites pukoensis* | living | Luhuitou |
| 53 | N2-2(1)-035 | *Porites pukoensis* | living | Luhuitou |
| 54 | N2-2(1)-052 | *Acropora pulchra* | living | Luhuitou |
| 55 | N2-2(1)-053 | *Acropora pulchra* | living | Luhuitou |
| 56 | N2-2(1)-054 | *Acropora tenuis* | living | Luhuitou |
| 57 | N2-2(1)-055 | *Acropora tenuis* | living | Luhuitou |
| 58 | N2-2(1)-064 | *Porites compressa* | living | Luhuitou |
| 59 | N2-2(1)-065 | *Porites compressa* | living | Luhuitou |
| 60 | N2-2(2)-001 | *Montipora monasteriata* | living | Luhuitou |
| 61 | N2-2(2)-002 | *Porites cyclindrica* | living | Luhuitou |
| 62 | N2-2(2)-003 | *Porites cyclindrica* | living | Luhuitou |
| 63 | N2-2(2)-006 | *Isopora brueggemanni* | living | Luhuitou |
| 64 | N2-2(2)-007 | *Isopora brueggemanni* | living | Luhuitou |
| 65 | N2-2(2)-008 | *Acropora valida* | living | Luhuitou |
| 66 | N2-2(2)-009 | *Acropora valida* | living | Luhuitou |
| 67 | N2-2(2)-060 | *Acropora millepora* | living | Luhuitou |
| 68 | N2-2(2)-061 | *Acropora millepora* | living | Luhuitou |
| 69 | NK-1-0008 | *Acropora cerealis* | fossil | Meiji |
| 70 | NK-1-0009 | *Acropora tenuis* | fossil | Meiji |
| 71 | NK-1-0063 | *Acropora hyacinthus* | fossil | Meiji |
| 72 | NK-1-0179 | *Porites lutea* | fossil | Meiji |
| 73 | NK-1-0188 | *Acropora florida* | fossil | Meiji |
| 74 | NK-1-0234 | *Astreopora myriophthalma* | fossil | Meiji |
| 75 | NK-1-0239 | *Pocillopora damicornis* | fossil | Meiji |
| 76 | NK-1-0242 | *Astreopora myriophthalma* | fossil | Meiji |
| 77 | NK-1-0303 | *Pocillopora damicornis* | fossil | Meiji |
| 78 | NK-1-0309 | *Pocillopora damicornis* | fossil | Meiji |
| 79 | NK-1-2652 | *Montipora sp.* | fossil | Meiji |
| 80 | NK-1-3416 | *Isopora brueggemanni* | fossil | Meiji |
| 81 | NK-1-3423 | *Porites lobata* | fossil | Meiji |
| 82 | NK-1-5833 | *Porites lutea* | fossil | Meiji |
| 83 | NK-1-6267 | *Porites lutea* | fossil | Meiji |

Table S2 Matrix of character states from thin section of *Acropora* species

|  |  | *A.robusta* | *A.abrotanoides* | *A.hyacinthus* | *A.tenuis* | *A.cerealis* | *A.florida* | *A.muricata* | *A.millepora* | *A.pulchra* | *A.valida* |
| --- | --- | --- | --- | --- | --- | --- | --- | --- | --- | --- | --- |
| 1 | axial corallites outer diameter | 2 | 2 | 0 | 1 | 0 | 1 | 1 | 2 | 2 | 1 |
| 2 | axial corallites inner diameter | 2 | 2 | 0 | 1 | 0 | 1 | 1 | 2 | 2 | 1 |
| 3 | axial corallites synapticular rings | 0 | 0 | 0 | 0 | 1 | 1 | 0 | 1 | 1 | 1 |
| 4 | axial corallites synapticular cavity filling | 0 | 0 | 1 | 1 | 1 | 1 | 0 | 1 | 1 | 1 |
| 5 | axial corallites primary septa length | 2 | 2 | 2 | 2 | 1 | 1 | 2 | 2 | 1 | 1 |
| 6 | axial corallites secondary septa cycle | 1 | 0 | 0 | 0 | 0 | 0 | 1 | 1 | 0 | 0 |
| 7 | axial corallites septa connectivity | 1 | 0 | 0 | 1 | 1 | 1 | 0 | 1 | 1 | 1 |
| 8 | axial corallites septa top swelling | 1 | 1 | 0 | 1 | 1 | 1 | 1 | 1 | 1 | 0 |
| 9 | axial corallites septa calcification lines width | 1 | 0 | 1 | 1 | 0 | 1 | 0 | 1 | 1 | 1 |
| 10 | axial corallites septa calcification lines curving | 1 | 1 | 0 | 0 | 1 | 1 | 1 | 0 | 0 | 0 |
| 11 | radial corallites synapticular rings | 0 | 0 | 0 | 0 | 1 | 1 | 0 | 0 | 0 | 1 |
| 12 | radial corallites primary septa cycle | 0 | 1 | 0 | 1 | 0 | 1 | 0 | 0 | 0 | 1 |
| 13 | radial corallites primary septa length | 1 | 0 | 1 | 0 | 0 | 1 | 1 | 2 | 2 | 2 |
| 14 | radial corallites directive septa directivity | 0 | 1 | 1 | 2 | 0 | 0 | 1 | 0 | 2 | 2 |
| 15 | coenosteum arrangement | 0 | 0 | 1 | 0 | 0 | 0 | 1 | 1 | 1 | 0 |
| 16 | coenosteum mesh size | 0 | 1 | 0 | 0 | 0 | 0 | 1 | 0 | 1 | 0 |
| 17 | coenosteum lateralbinding | 1 | 1 | 1 | 1 | 1 | 1 | 0 | 1 | 0 | 0 |
| 18 | coenosteum marginal palisading arrangment | 0 | 0 | 1 | 1 | 1 | 1 | 0 | 0 | 0 | 0 |
